# The Complete Mitochondrial Genome of *Thymus mongolicus* and Its Phylogenetic Relationship with Lamiaceae Species

**DOI:** 10.3390/biom15030343

**Published:** 2025-02-27

**Authors:** Na Na, Zinian Wu, Zhiyong Wang, Yanting Yang, Chunyu Tian, Lin Zhu, Taiyou Ou, Xiaofei Chen, Hongyan Xia, Zhiyong Li

**Affiliations:** 1 Institute of Grassland Research, Chinese Academy of Agricultural Sciences, Hohhot 010011, China; 13684752695@163.com (N.N.);; 2 Key Laboratory of Grassland Resources and Utilization of Ministry of Agriculture, Hohhot 010018, China; 3 Inner Mongolia General Station of Seed and Seedling of Forestry and Grassland, Hohhot 010021, China

**Keywords:** Lamiaceae, mitochondrial genome, phylogenetic analysis, RNA editing, *Thymus mongolicus*

## Abstract

*Thymus mongolicus* (Lamiaceae) is a plant commonly found throughout China, in which it is widely used in chemical products for daily use, traditional medicinal preparations, ecological management, and cooking. In this study, we have assembled and annotated for the first time the entire mitochondrial genome (mitogenome) of *T. mongolicus*. The mitochondrial genome of *T. mongolicus* is composed in a monocyclic structure, with an overall size of 450,543 base pairs (bp) and a GC composition of 45.63%. It contains 32 unique protein-encoding genes. The repetitive sequences of the *T. mongolicus* mitogenome include 165 forward repetitive sequences and 200 palindromic repetitive sequences, in addition to 88 simple sequence repeats, of which tetramers accounted for the highest proportion (40.91%). An analysis of the mitogenome codons revealed that synonymous codons generally end with A/U. With the exception of nad4L, which uses ACG/ATG as an initiation codon, all other genes begin with the ATG start codon. Codon analysis of the mitogenome also showed that leucine (909) are the most abundant amino acid, while tryptophan (134) are the least prevalent. In total, 374 RNA editing sites were detected. Moreover, 180 homologous segments totaling 105,901 bp were found when the mitochondrial and chloroplast genomes of *T. mongolicus* were compared. Phylogenetic analysis further indicated that *T. mongolicus* is most closely related to *Prunella vulgaris* in the Lamiaceae family. Our findings offer important genetic insights for further research on this Lamiaceae species. To the best of our knowledge, this study is the first description of the entire mitogenome of *T. mongolicus*.

## 1. Introduction

*Thymus mongolicus* is a plant within the Lamiaceae family, the natural distribution of which extends from Siberia to China. It is a sub-shrub that predominantly thrives in the temperate biome [1,2]. *T. mongolicus* is a multi-purpose plant with extensive developmental value, covering aspects such as medicine, nutrition, cosmetics, and seasoning. Its rich chemical composition, including terpenoids and flavonoids, indicates its remarkable potential in medicinal and healthcare applications. It also serves as a natural flavoring agent to improve taste and enhance the nutritional value of food [3,4]. Moreover, *T. mongolicus* essential oils have been established to have antioxidant and antibacterial properties and are widely used in cosmetics and skincare products that contribute to maintaining skin health, combatting aging, and relieving acne [5]. In addition, *T. mongolicus* is commonly used as a seasoning in different types of cooking, including lamb, soups, and roasted dishes, imparting a unique flavor [6]. *T. mongolicus* also plays an important ecological role in that its short creeping stems can develop strong root systems that can contribute to preventing soil erosion [7]. Consequently, the thyme cultivation industry is considered to have considerable developmental potential, which can provide new directions for the promotion of diversified agricultural production models.

Mitochondria, the primary energy providers in eukaryotic cells, are semi-autonomous organelles that contain independent genetic materials and regulatory mechanisms, which are essential for cell energy metabolism [8]. Mitochondria are involved in the regulation of key metabolic processes, such as cell differentiation, apoptosis, growth, and division [9], as well as play prominent roles with respect to stress tolerance, plant growth vigor, and male sterility linked to the cytoplasm [10]. The majority of plant mitochondrial genomes differ significantly in their structure and content, nucleotide substitution rates, and repetitive sequences [11,12], resulting in the occurrence of complex structural types, such as circular, branched, and reticulated mitochondrial genomes, with genome lengths ranging from 22 kb in *Avicennia marina* to 11.7 Mb in *Larix sibirica* [13,14]. Moreover, these genomes are characterized by their high frequency of inversions and recombination events, frequent structural rearrangements, low rate of synonymous mutations [15], the presence of large numbers of non-coding sequences, the prominent occurrence of repetitive elements, and a wide range of RNA editing sites [12]. These features accordingly contribute to the general instability of the mitochondrial DNA structure, as well as a complex assembly process [16]. Consequently, compared with that of chloroplast DNA, progress in the sequencing of mitochondrial DNA has tended to advance somewhat less rapidly [17]. Nevertheless, given the recent rapid advances in sequencing technologies and assembly techniques, the number of fully sequenced mitogenomes has steadily increased. The National Center for Biotechnology Information (NCBI) database (https://www.ncbi.nlm.nih.gov/ (accessed on 25 October 2024)) contains the sequences of some 16,700 complete chloroplast genomes, whereas only approximately 800 complete plant mitogenome sequences are currently available. Within the family Lamiaceae, the mitochondrial genomes of plants, including *Lavandula angustifolia* Mill [18], *Haberlea rhodopensis* [19], *Boea hygrometrica* [20], *Coptis chinensis* [21], *Piper betle* [22], *Physcomitrella patens* [23], *Nymphaea colorata* [24], *Apium graveolens* [25], and *Actinidia chinensis* [26], have been assembled and published. However, although the mitogenomes of a number of plants have been sequenced and analyzed, there has, to date, been comparatively limited research on the mitogenome of *T. mongolicus*.

In this research, we assembled the complete mitochondrial genome of *T. mongolicus* and analyzed various genomic characteristics, such as its GC composition, repetitive sequences, RNA editing sites, codon usage patterns, and phylogenetic relationships. Furthermore, we explored the mitochondrial structure and examined the genetic exchange between the chloroplast and mitochondrial genomes. The outcomes of this research will enhance our understanding of the mitochondrial genome and genetic variability in *T. mongolicus*, providing a strong basis for future investigations into mitochondrial genomes.

## 2. Materials and Methods

### 2.1. DNA Isolation, Sequencing, and Genome Assembly

Fresh seedlings of *T. mongolicus* were harvested and preserved at the National Perennial Forage Germplasm Resource Nursery of the Institute of Grassland Research, Chinese Academy of Agricultural Sciences, in Hohhot, Inner Mongolia, China (40.57° N, 111.93° E). The identity of the plant specimens was verified by Zinian Wu. Prior to use, the samples were stored at −80 °C. Genomic DNA was isolated from fresh leaf tissue using the CTAB method [27] and Qiagen Blood & Cell Culture DNA Kit (Cat. no. 13323). The mitogenome of *T. mongolicus* was sequenced by the NovaSeq 6000 (Illumina, San Diego, CA, USA) and Nanopore PromethION (Oxford Nanopore Technologies, Ningjing, China) platforms. The mitogenomic sequence of *T. mongolicus* was obtained using minimap2 (v2.1) [28] to align the Nanopore sequencing data to the reference plant mitochondrial core gene sequences (reference link: https://github.com/xul962464/plant_mt_ref_gene (accessed on 17 November 2024)). These data were corrected using Canu (v1.4) [29]. The Illumina sequencing data were mapped to the Nanopore corrected sequence using bowtie2 (v2.3.5.1) [30] and Unicycler (v0.4.8) [31] was employed to assemble these data using default parameters. Finally, Bandage (v0.8.1) [32] was used to visualize the assembly outcomes, with manual modifications applied when required.

### 2.2. Mitogenome Annotation

For the purposes of annotating the protein-coding genes (PCGs) in the mitochondrial genome of *T. mongolicus*, we used the mitochondrial genome of *Prunella vulgaris* (NC081933) as a reference, and Geseq software (v2.03) [33] and PMGA were used for genome annotation [34]. tRNAscan-SE (v2.0.12) [35], with default settings, was used to verify the tRNA and rRNA genes, and ORFfinder was applied for the analysis of open reading frames larger than 300 bp (https://www.ncbi.nlm.nih.gov/orffinder/ (accessed on 17 November 2024)). A circular mitochondrial map was drawn using Organellar Genome DRAW (v1.3.1) [36]. The annotation information thus obtained for the *T. mongolicus* mitogenome has been uploaded to the NCBI with the accession number PP723039.

### 2.3. Identification of Repeat Sequences

As part of our characterization of the *T. mongolicus* mitogenome, we examined simple, tandem, and scattered repeats. Searches for simple sequence repeats (SSRs) [37] were performed with the following minimum repeat unit thresholds: 10 for mononucleotides, 5 for dinucleotides, 4 for trinucleotides, and 3 for both four-nucleotide and five-nucleotide repeats, which were identified using MISA software (v2.1) [38]. Tandem repeats were identified using the Tandem Repeats Finder (v4.10.0) [39] with the following parameters: 2, 7, 7, 80, 10, 50, 2000, -f, -d, and -m. In addition, dispersed repeats longer than 30 bp, including forward, reverse, palindromic, and complementary repeats, were detected using the REPuter online tool [40], with a Hamming distance of 3 and an e-value threshold of 1 × 10^−5^.

### 2.4. Analysis and Prediction of Codon Usage Patterns and RNA Editing Regions

The CodonW software (v1.4.4) [41] was used to analyze the relative synonymous codon usage (RSCU) of the PCGs. To predict RNA editing sites, PCGs from the mitochondrial genome were input as text files into the Deepred-mt tool [42]. Only predictions with a probability greater than 0.9, derived from a convolutional neural network model, were considered trustworthy.

### 2.5. Identification of Chloroplast-Derived Mitochondrial Sequences

The chloroplast genome of *T. mongolicus* was reconstructed with GetOrganelle software (v1.7.0) [43] and then annotated using CPGAVAS2 (v0.03) software [44]. BLASTN software (v2.13.0) [45] was used to analyze homologous fragments, and TB tools (v2.056) [46] software was used to visualize gene transfer from the chloroplast to the mitochondrion.

### 2.6. Phylogenetic Analysis of the T. mongolicus Mitochondrial Genome

To establish the phylogenetic placement of *T. mongolicus*, we obtained the mitochondrial genome sequences of 12 Lamiaceae species (Table S1) featuring 16 conserved mitochondrial protein-coding genes from the NCBI repository, with *Medicago truncatula* and *Arabidopsis thaliana* serving as outgroup species. The sequences, including the newly sequenced *T. mongolicus* mitochondrial DNA, were employed to construct a phylogenetic tree. All nucleotide sequences were aligned using MAFFT (v7.505) software [47] with default parameters. To generate phylogenetic trees, we used the maximum likelihood (ML) and Bayesian inference (BI) methods, and ModelFinder was used to identify the best-fitting model [48]. The maximum likelihood tree with 1000 replicates was constructed using IQ-TREE (v2.0.3) [49] and GTR + F + I + R3 models, whereas a Bayesian tree was generated using MrBayes (v3.2) [50] with the GTR + F + I + G4 model.

## 3. Results

### 3.1. Genomic Characteristics of the T. mongolicus Mitogenome

**Figure 1:**
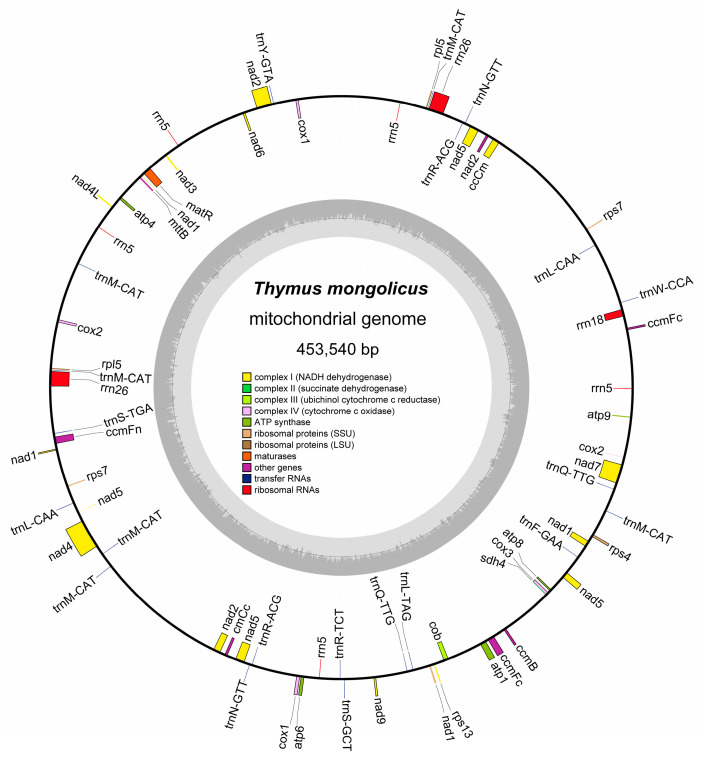
The complete mitochondrial genome of *Thymus mongolicus* is represented in a circular diagram. In the figure, the genes of the outer ring showed positive transcription, while the genes of the inner ring showed reverse transcription. In the inner ring of the figure, the GC content was highlighted in an intuitive carbon gray tone, and different functional gene categories were color-coded.

By combining Nanopore and Illumina sequencing techniques, we successfully reconstructed the mitochondrial genome of *T. mongolicus*, for which we obtained a ring structure with typical terrestrial plant genome characteristics. The overall length of the mitochondrial genome is 453,540 bp, which fully demonstrates the integrity and robustness of the mitochondrial genome of *T. mongolicus* (Figure 1). After in-depth annotation of the assembly results, we identified 32 PCGs with a total coverage length of 29,367 bp and a GC content of 42.25%. In addition, the mitogenome contains multiple non-core genes, reflecting the diversity and complexity of the mitochondria. Specifically, the total length and GC content of the eight rRNA genes were 9833 bp and 49.93%, respectively, whereas the corresponding values for the 21 tRNA genes were 1599 bp and 51.84%. We also discovered two pseudogenes.

The identified 32 PCGs accounted for 6.47% of the total length of the mitochondrial genome, namely 29,367 bp. These genes include three cytochrome c oxidase genes (*cox1*, *cox2*, and *cox3*), one maturase gene (*matR*), one ubiquinol–cytochrome *c* reductase gene (*cob*), 11 NADH dehydrogenase genes, two large ribosomal protein subunit genes (*rpl5*), three small ribosomal protein subunit genes (*rps13* and *rps7*), and one transport membrane protein gene (*mttB*). Notably, *ccmC*, *nad2*, *nad5*, *rpl5*, and *rps7* each have two copies. Furthermore, we identified eight rRNA and 21 tRNA genes, accounting for 2.17% and 0.35% of the total mitogenome length, respectively. Among the rRNA genes, there are two copies of *rrn26*, whereas *rrn5* has five, and among the tRNA genes, *trnM-CAT* has six copies, whereas *trnL-CAA*, *trnN-GTT*, *trnQ-TTG*, and *trnR-ACG* each have two copies (Table 1).

The mitogenome of *T. mongolicus* also features 10 intron-containing genes. Specifically, *nad1*, *nad2* (two copies), and *nad5* (two copies) contain four introns; *nad4* and *nad7* have three introns; *ccmFc* has two; and both *cox1* and *cox2* have a single intron. Moreover, we found that the exonic and intronic structures of these genes have unique characteristics. For example, *nad1* contains five exons and four introns, whereas *nad2* and *nad5* have ten exons and eight introns, and *nad7* has four exons and three introns (Table S2).

### 3.2. Examination of Repetitive Sequences in the T. mongolicus Mitochondrial Genome

**Figure 2:**
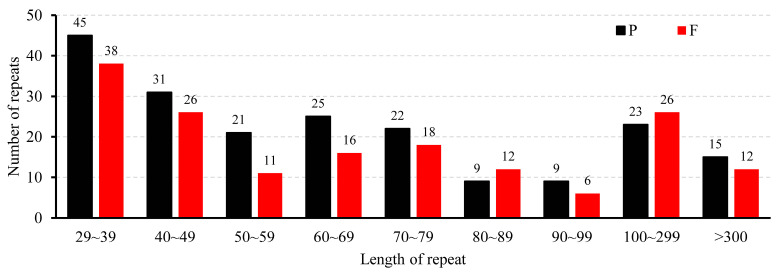
The length distribution of dispersed repeats in the Thymus mongolicus mitogenome is categorized into the following types: P for palindromic, F for forward.

In this research, we conducted an extensive examination of the scattered repetitive sequences within the *T. mongolicus* mitochondrial genome. In total, we identified 365 dispersed repetitive sequences with a combined length of 125,312 bp, with all these having lengths of at least 29 bp. Among these sequences, we detected 165 (45%) forward repeats and 200 (55%) palindrome repeats, although we failed to identify any reverse or complementary sequences. With respect to the length distribution of these repetitive sequences, we found that the majority (338) lie within the range from 29 to 300 bp, representing 93% of the overall total. Among the remaining repetitive sequences, 16 (4%) were longer than 1000 bp, notable among which was a forward repeat of 42,019 bp, which constitutes 33.53% of the total length of the dispersed repetitive sequences and 9.26% of the entire mitogenome. With respect to the number of repetitions, sequences with lengths of between 29 and 39 bp were found to be the most frequent type, with 83 occurrences, including 45 palindromic and 38 forward repeats. In contrast, sequences with lengths of 90–99 bp are the least frequent (Figure 2, Table S3).

**Figure 3:**
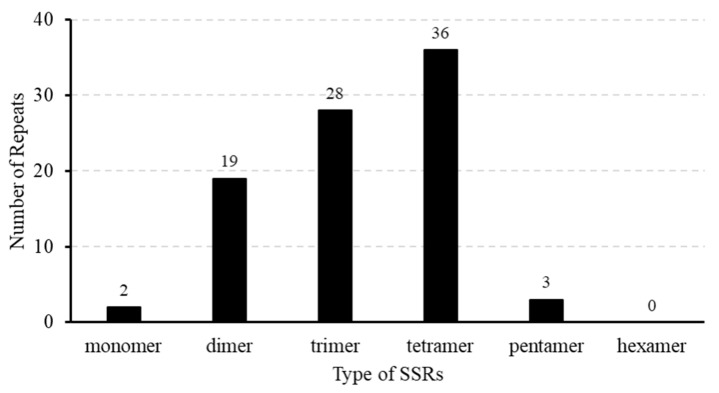
The distribution of simple sequence motifs (SSRs) in the *Thymus mongolicus* mitogenome.

We also detected 88 SSRs in the mitogenome of *T. mongolicus*, with an overall length of 1044 bp, among which, 2 (2.27%), 19 (21.59%), 28 (31.82%), 36 (40.91%), and 3 (3.41%) are mononucleotide, dinucleotide, trinucleotide, tetranucleotide, and pentanucleotide repeat sequences, respectively (Figure 3, Table S4). The repeating units of the SSRs included one type of monomer (A/T), two types of dimer, six types of trimer, fourteen tetramers, and three types of pentamer. The majority of SSRs (82) are located in the intergenic regions (IGS), with *nad5* containing the highest number of SSRs (35) and *cox2* containing the most diverse types of repeats. The remaining six SSRs are distributed in IGS, with four located between the start factor and *ccmFc* and the other two between the *cox2*-*atp9* and *atp9*-*rrn5* genes. Additionally, two SSRs were found in the exonic regions of *rrn18* (TTTC sequence) and *nad1* (Table S5).

**Figure 4:**
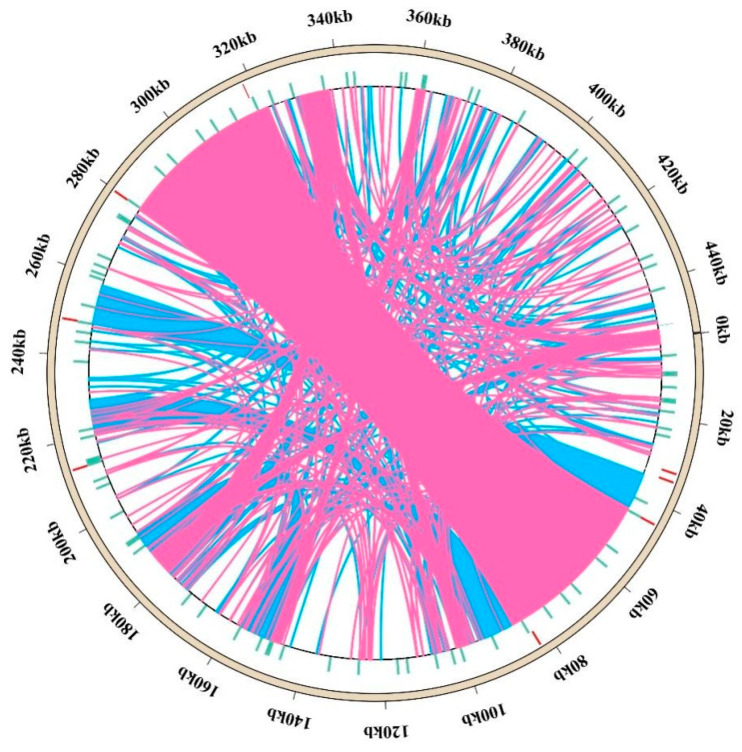
Distribution of the dispersed repeats in the *Thymus mongolicus* mitogenome. The outermost circle depicts the mitogenome sequence. Simple sequence, tandem, and dispersed repeats are represented inside the mitogenome circle. The pink and blue arcs represent 165 forward and 200 palindromic repeats, respectively.

Furthermore, we identified 10 tandem repeat sequences with lengths of between 32 and 67 bp that are evenly distributed within the IGS regions of the *T. mongolicus* mitogenome, the total length of which is 541 bp, with a similarity matching greater than 80% (Table S6). Collectively, the SSRs (88), tandem repeats (10), and dispersed repeats (365) have an overall length of 126,597 base pairs and account for 27.91% of the mitogenome (Figure 4).

### 3.3. Examination of Codon Preferences in PCGs of the T. mongolicus Mitogenome

**Figure 5:**
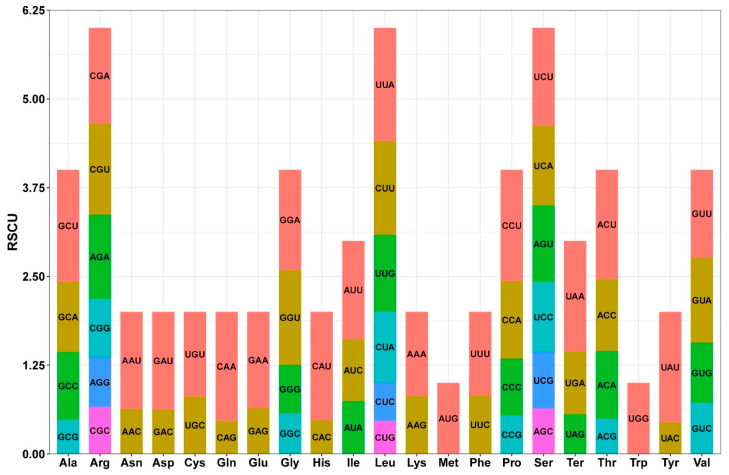
Relative synonymous codon usage (RSCU) value of PCGs in *T. mongolicus* mitochondrial genome. The x-axis shows the codon family. The y-axis represents the frequency of use. Codons encoding the same amino acid are distinguished by different colors.

Among the 32 PCGs detected in the complete mitogenome of *T. mongolicus*, we identified 8199 codons (Table S7). The mtDNA of *T. mongolicus* encodes 20 amino acids, for which we identified 61 different types of codons. RSCU analysis revealed that synonymous codons in the mitogenome of *T. mongolicus* preferentially ended with A/U (Figure 5). The majority of the PCGs were found to have a typical ATG start codon, with an exception in this regard being nad4L, which uses the alternative start codon ACG (Table S8). Three stop codons were identified, namely, TAG, TAA, and TGA. Each amino acid corresponds to at least one codon, up to six codons. In terms of the frequency of occurrence, leucine was found to be the most common amino acid in the PCGs, occurring 909 times (11.05%), followed by serine, which appeared 754 times (9.16%), and arginine, which occurred 467 times (5.68%). In contrast, methionine and tryptophan were found to be the least common, appearing only 237 (2.88%) and 134 (1.61%) times, respectively. Moreover, UUU was detected 340 times in phenylalanine, corresponding to an RSCU value of 1.18, whereas UUA was detected 242 times in leucine, yielding an RSCU value of 1.6. The least used codon was the TAG stop codon, which was used six times with an RSCU value of 0.6.

### 3.4. Prediction and Analysis of RNA Editing Sites in the T. mongolicus Mitogenome

**Figure 6:**
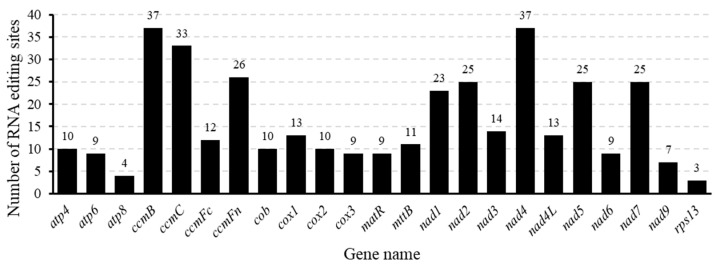
The spread of RNA editing sites across the mitochondrial protein-coding genes of *Thymus mongolicus*.

In plants, RNA editing occurs primarily within organelles, including the mitochondria and chloroplasts [51], in which it plays a pivotal role in their specific functions. Consequently, we examined the locations of RNA editing sites within 23 distinct PCGs found in the mitogenome of *T. mongolicus*. Among the mitochondrial genes, *ccmB* and *nad4* were identified as containing the highest proportion of RNA editing sites, each with 37 (Figure 6), whereas *rps13* has only three such sites. Collectively, 374 RNA editing sites were identified to be associated with seven types of base change, with a C-to-U change being overwhelmingly predominant (*n* = 368, 98.4%). Other changes occur at considerably lower frequencies, with each occurring only a single time. Further analysis of the RNA editing sites revealed that 43 codons were altered. Specifically, editing primarily occurred at the second base of the codon (229, 61.2%), followed by the first base (119, 31.8%), whereas changes at the third base were the least frequent (21, 5.6%). In one codon, we identified a change in all three bases (CUC to UCU), and two-base changes were detected in a further four. Notably, codons in both *cob* and *cox2* were found to have undergone RNA editing, converting them to start codons (AUG), whereas codons in *nad5* and *atp4* were edited to stop codons (UAG or UAA) (Table S9). It can be speculated that these editing events may be important with respect to the regulation of mitochondrial protein synthesis and function.

**Figure 7:**
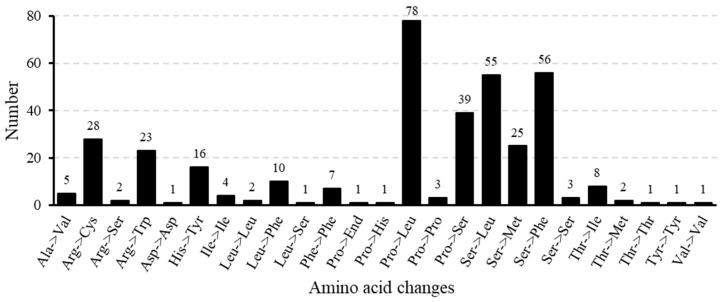
Frequency of amino acid alterations resulting from RNA editing in the *Thymus mongolicus* mitogenome sequence.

Collectively, we established that RNA editing processes have been implicated in 25 amino acid changes, including nine synonymous alterations. Among these, the most frequent change was the conversion of proline to leucine (Figure 7). These findings tend to imply that the scope and magnitude of RNA editing in the *T. mongolicus* mitogenome are relatively limited, thereby indicating a propensity for stability and conservation.

### 3.5. DNA Migration from Chloroplast to Mitochondria

**Figure 8:**
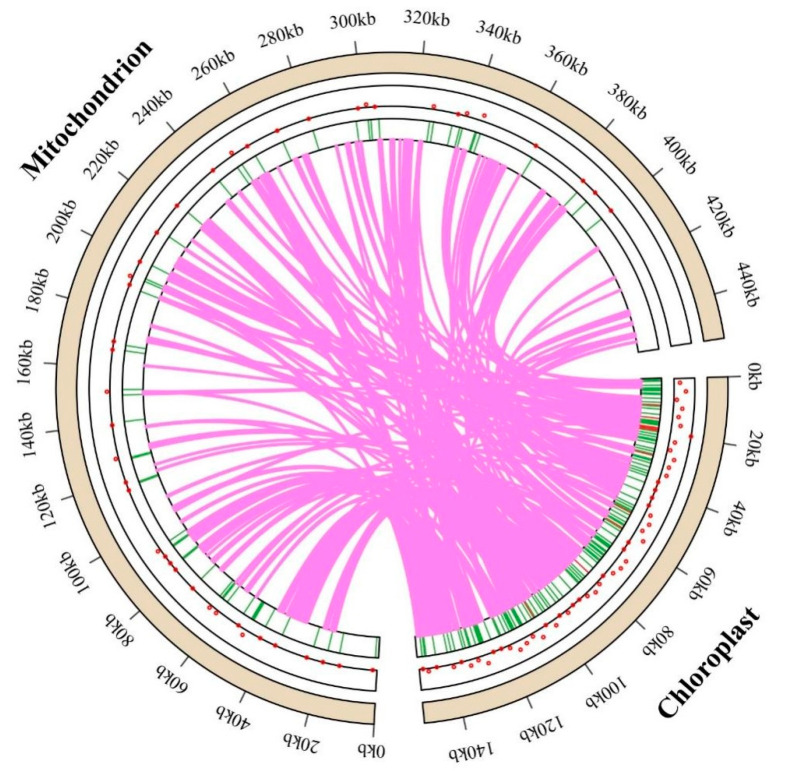
Gene exchange events between the chloroplast and mitochondrial genomes of *Thymus mongolicus*, with pink lines representing the pathways through which chloroplast-derived sequences were incorporated into the mitogenome. Red dots and green lines mark the locations of the corresponding similar sequences.

Given that the mitogenome (453,540 bp) of *T. mongolicus* is approximately three times the size of the chloroplast genome (151,787 bp), the distribution range of the former is somewhat wider than that of the latter (Figure 8). The mitochondrial genome of *T. mongolicus* contains 180 putative chloroplast-derived fragments that we believe may have been acquired through gene transfer based on the sequence similarity between the mitochondrial and chloroplast genomes (Figure 8, Table S10). The total length of these fragments is 105,901 bp, which accounts for 69.77% of the chloroplast genome. Among these fragments, the longest comprised *ycf15*, *trnL-CAA*, *ndhB*, *ndhB* (intron), and *rps12* (intron), as well as *ycf15*, *ndhB* (intron), *ndhB*, and *trnL-CAA*, which we assume were incorporated from the chloroplast into the mitochondrial intergenic spacer (IGS) region (*trnL-CAA*). The combined length of these fragments is 8786 bp, showing an exact match of 98.976%. Additional examination of related sequences showed that the chloroplast genome of *T. mongolicus* harbors several genes that were fully integrated into the mitogenome, including one ribosomal PCG (*rps7*), two NADH dehydrogenase genes (*ndhF*), three tRNA genes (*trnS-UGA*, *trnQ-UUG*, *trnS-GGA*), and sixteen rRNA genes (*rrn16* and *rrn23*). These genes were transferred to the mitogenome in the *rps7*, *trnS-UGA*, *trnQ-UUG*, *trnS-GCU*, *rrn18*, and *rrn26* regions. The IGS of the mitogenome contains the majority of the sequences that were transferred from the chloroplast genome.

### 3.6. Phylogenetic Analysis of the T. mongolicus Mitogenome

**Figure 9:**
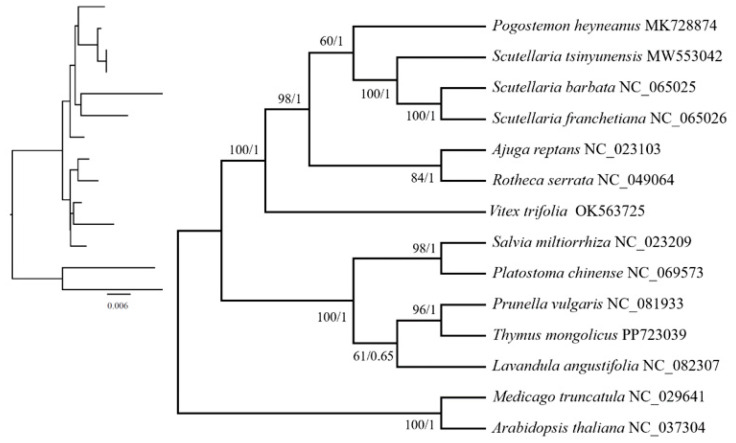
A phylogenetic tree of the mitochondrial genomes of 12 Lamiaceae species and two outgroups constructed based on 16 conserved protein-coding genes. The bootstrap values from the maximum likelihood and Bayesian posterior probability methods are shown at each node. The left branch represents the genetic relationship and evolutionary distance among species (Phylogram), and the nodes of the simple tree structure on the right side represent common ancestors (Cladogram).

We constructed a phylogenetic tree based on 16 conserved protein-coding genes (PCGs) from the mitochondrial genomes of 12 species in the Lamiaceae family, employing the maximum likelihood (ML) method to explore the evolutionary connections between *T. mongolicus* and its closely related species (Figure 9). A total of five nodes were identified, and excluding the outgroup, the other four nodes revealed that the species can be divided into three categories, including the 12 Lamiaceae species. The first category includes *Rotheca*, *Ajuga*, *Scutellaria*, and *Pogostemon*; the second consists of *Vitex*; and the third comprises *Salvia*, *Platostoma*, *Prunella*, *Thymus*, and *Lavandula*. The results show that *T. mongolicus* is a member of the genus *Thymus* within the Lamiaceae family and forms a close clade with *P. vulgaris*, indicating a close phylogenetic relationship.

## 4. Discussion

### 4.1. Characteristics of the T. mongolicus Mitogenome

The mitochondrial genomes of a majority of plants are between 200 and 700 kb in length, whereas the smallest is that *Avicennia marina* (22 kb) [14] and the largest is that of *Larix sibirica* (11.7 Mb) [13], and species typically differ with respect to the structure of their mitochondrial genomes [52]. In the present study, we established that the mitochondrial genome of *T. mongolicus* has a single-loop structure with a total length of 453,540 bp, which is larger than that of other Lamiaceae species, such as *Pogostemon heyneanus* (380,655 bp), *Scutellaria barbata* (372,525 bp), *Arabidopsis thaliana* (367,808 bp), *Lavandula angustifolia* (355,345 bp), *S. franchetiana* (354,302 bp), *S. tsinyunensis* (354,073 bp), *Ajuga reptans* (352,069 bp), and *P. vulgaris* (297,777 bp), although is smaller than those of *Salvia miltiorrhiza* (499,236 bp), *Platostoma chinense* (494,599 bp), and *Rotheca serrata* (482,114 bp) (Table S1). The size of the mitochondrial genome of *T. mongolicus* differs significantly from that of other Lamiaceae species, indicating that it is a species with a relatively large mitochondrial genome in Lamiaceae, which may be related to species differences [53]. Conversely, however, the GC content of the *T. mongolicus* mitogenome (45.63%) is relatively consistent with that of other species within this family, with values of 43.92%, 44.39%, 45.26%, 45.29%, 45.19%, 45.10%, 45.14%, 44.21%, 45.62%, 44.67%, and 45.54% being reported for *P. vulgaris*, *S. miltiorrhiza*, *S. tsinyunensis*, *S. franchetiana*, *S. barbata*, *A. reptans*, *L. angustifolia*, *P. chinense*, *V. trifolia*, *P. heyneanus*, and *R. serrata*, respectively. However, although the mitochondrial genome of thyme is relatively conserved with respect to GC content, it differs to a more considerable extent in terms of genome size, composition, and structure, particularly its size, which is consistent with the findings of previous studies [54,55]. The diversity of this structure is considered to be indicative of the adaptability of the mitochondrial genome, which enables it to respond effectively to changes in both intra- and extracellular environments [12]. Moreover, the consistency of GC content further supports the view that the GC contents of genomes have remained relatively stable during the course of higher plant evolution.

However, despite the considerable variation in the size of plant mitogenomes, the number of mitochondrial genes has tended to remain relatively conserved among terrestrial plants [56,57]. We established that the mitogenome of *T. mongolicus* contains 61 genes, comprising 32 PCGs, 8 rRNA genes, and 21 tRNA genes (Table S8). Furthermore, given that codons carry important identification and transition information in seed-bearing plants, their usage bias is affected by species-specific variation, which plays a key role in shaping genetic characteristics [58]. Accordingly, we also analyzed codon usage bias among the 32 PCGs. Notably, we established that each amino acid in the mitogenome is represented by at least one kind of codon (methionine and tryptophan), with a maximum of six kind possible codons (leucine, serine, and arginine). The usage frequency of codons for each amino acid also varies, indicating that codon usage may be associated with the gene expression [59,60]. Moreover, we observed a marked preference for synonymous codons terminating in A/U within the mitogenome of *T. mongolicus*. The codon usage patterns characterized in this study are, however, generally consistent with those previously documented for the mitogenomes of other species [61,62].

### 4.2. Repeat Sequences Within the T. mongolicus Mitogenome

Repetitive sequences are essential for preserving the structural integrity of non-coding regions in plant mitogenomes [63], in which they contribute to genome rearrangement, inversion, insertion, and deletion [64]. The mitogenome structure varies significantly among different plant species [65], and in the mitochondrial genome of *T. mongolicus* A, we identified a dispersed repeat sequence of 42,019 base pairs, which we speculate could be closely associated with the rearrangement and recombination of the genome [66]. The differences in plant mitogenome sizes are mainly attributable to variations in the length of repeat sequences [53], and longer repeats are more likely to be involved in genome rearrangement, leading to frequent rearrangement events [67]. Mitogenomes rich in SSRs [68] are often characterized by their relatively large size, gene duplication, structural changes, and genomic diversity [69], and these repeats can make a valuable contribution to facilitating species identification and evaluating evolutionary relationships [70]. Our findings in this study revealed that SSRs in the mitogenome of *T. mongolicus* mainly comprise tetranucleotide polymers, which is consistent with the findings reported for other plants [52]. The number and type of repeat sequences identified in the *T. mongolicus* mitogenome are similar to those reported for *S. miltiorrhiza*. Notably, however, unlike the mitogenome of *P. vulgaris,* which contains tandem repeats [71], those of *T. mongolicus* and *S. miltiorrhiza* lack these features. The differences in these repeat sequences are consistent with their mitochondrial genome size, which may be related to the amplification and deletion of elements [72].

### 4.3. RNA Editing in the T. mongolicus Mitogenome

In general, there are more RNA editing sites in plant mitochondrial genomes than in chloroplast genomes. In our research of the *T. mongolicus* mitogenome, we detected 374 RNA editing sites in total, which is less than those in *P. vulgaris*. Both *T. mongolicus* and *P. vulgaris* contain C-to-U RNA editing sites. A large proportion of RNA editing occurrences in plant organelles are caused by site-specific C-to-U transformations [73]. In addition, we found that the frequency of changes occurring at the third position of the codons at RNA editing sites within the mitogenome was relatively low, which is consistent with previous findings [74,75]. Furthermore, non-synonymous changes outnumbered synonymous changes, which may indicate that gene mutations have occurred during evolution [52]. We found a significant proportion of RNA editing sites were at the second codon position, a pattern that mirrors the pattern observed in sugarcane, in which approximately 61.49% of the editing sites also occur at this position [2]. This pattern may reflect the selective pressure on the RNA editing mechanism in mitogenomes during evolution, thereby further influencing gene function and adaptability. A more extensive understanding of the intricate mechanisms that drive plant environmental adaptation, gene expression regulation, and evolutionary processes can be gained via a comprehensive analysis of these editing sites.

### 4.4. Chloroplast-Derived Sequences in the T. mongolicus Mitogenome

Chloroplasts and mitochondria are vital organelles in plant cells, playing key roles in processes such as photosynthesis and cellular respiration, respectively [76]. The exchange of genetic material between these organelles is a complex and significant biological phenomenon that not only influences the function of organelles but also plays important roles in the evolution and adaptation of organisms [77,78], leading to diverse structural changes within the mitochondrial genome of plants. Our findings in this study provide evidence to indicate that fragments of chloroplast genes have been transferred and integrated into the mitogenome of *T. mongolicus* in a dynamic rather than a random process.

### 4.5. Phylogenetic Relationships of the T. mongolicus Mitogenome

Labiatae is one of the most widely distributed families in the world, containing about 200 genera and more than 3500 species [79]. While traditional classification methods mainly rely on morphological features for delineation, this study constructed a phylogenetic tree based on shared protein genes in the mitochondrial genome. The results of the analyses showed that the outgroups of Labiatae differed significantly from one species to another and that these plants shared a common ancestor. In the phylogenetic analyses of this study, *T. mongolicus* and *P. vulgaris* formed one branch, and together with *L. angustifolia*, they formed another branch. This result is more consistent with the phylogenetic relationship of chloroplasts of *T. mongolicus* [1]. In addition, the phylogenetic relationships of *S. tsinyunensis*, *S. franchetiana*, *S. barbata*, *P. heyneanus*, *A. reptans*, *V. trifolia*, and *P. chinense*, etc., are in better agreement with the results of previous phylogenetic studies on the mitochondrial genomes of *L. angustifolia* [57] and *P. vulgaris* [71], suggesting that these phylogenetic relationships have a high degree of reliability. Currently, the mitochondrial genome of *T. mongolicus* has not been reported; therefore, this study further explores the phylogenetic relationships of *T. mongolicus* from the perspective of the mitochondrial genome, providing new insights into the evolutionary studies of this taxon.

## 5. Conclusions

In this study, we reconstructed the complete mitochondrial genome of *T. mongolicus*, which spans 453,540 bp and has a GC content of 45.63%. The mitogenome contains 61 genes, among which there are 32 PCGs, 21 tRNA genes, and 8 rRNA genes. We identified 88 SSR loci and 374 RNA editing sites, with a notable preference for A/T endings. A homologous sequence alignment between the mitochondrial and chloroplast genomes revealed evidence of chloroplast gene transfer events. Furthermore, ML-based phylogenetic assessment revealed a strong connection between *T. mongolicus* and *P. vulgaris*. This complete sequence of the mitochondrial genome of *T. mongolicus* provides valuable insights into this plant’s genetic evolution and will serve as a foundation for future genetic breeding efforts.

## Figures and Tables

**Table 1:** Gene composition of the mitochondrial genome of *Thymus mongolicus*.

Group of Genes	Gene Name
ATP synthase	*atp1*, *atp4*, *atp6*, *atp8*, *atp9*
Cytochrome *c* biogenesis	*ccmB*, *ccmC* (2), *ccmFc* **, *ccmFn*
Ubiquinol–cytochrome *c* reductase	*Cob*
Cytochrome *c* oxidase	*cox1* *, *cox2* *, *cox3*
Maturase	*matR*
Transport membrane protein	*mttB*
NADH dehydrogenase	*nad1* ****, *nad2* **** (2), *nad3*, *nad4* ***, *nad4L*, *nad5* **** (2), *nad6*, *nad7* ***, *nad9*
Ribosomal protein (LSU)	*rpl5* (2)
Ribosomal protein (SSU)	#*rps4*, *rps13*, *rps7* (2)
Succinate dehydrogenase	#*sdh4*
rRNAs	*rrn18*, *rrn26* (2), *rrn5* (5)
tRNAs	*trnF-GAA*, *trnL-CAA* (2), *trnL-TAG*, *trnM-CAT* (6), *trnN-GTT* (2), *trnQ-TTG* (2), *trnR-ACG* (2), *trnR-TCT*, *trnS-GCT*, *trnS-TGA*, *trnW-CCA*, *trnY-GTA*

Notes: *, introns, the number of the “*” means the number of the introns; # Gene, pseudogene; (n), number of gene copies.

## Data Availability

The genome sequence data that support the findings of this study are openly available in GenBank of NCBI at (https://www.ncbi.nlm.nih.gov/ (accessed on 8 December 2024)). The accession number is PP723039.

## Supplementary Materials

The following supporting information can be downloaded at: https://www.mdpi.com/article/10.3390/biom15030343/s1, Table S1: GenBank accession numbers of mitochondrial genomes of 14 plant species used in this study; Table S2: Length of introns and exons in *T. mongolicus mitogenome* mitochondrial genome; Table S3: Scattered repeats in the mitochondrial genome of *T. mongolicus mitogenome*; Table S4: Spatial distribution of SSRs in the mitochondrial genome of *T. mongolicus mitogenome*; Table S5: Microsatellite repeats in the mitochondrial genome of *T. mongolicus mitogenome*; Table S6: Pattern of tandem repeat distribution in the mitochondrial genome of *T. mongolicus mitogenome*; Table S7: Frequency of codons in the protein-coding genes of the *T. mongolicus mitogenome*; Table S8: Information on the start and stop codons of mitochondrial genes in *T. mongolicus mitogenome*; Table S9: The table provides details on RNA editing sites in the mitochondrial genome of *T. mongolicus mitogenome*, with ** marking the start codon acquisition and * indicating the stop codon acquisition; Table S10: In *T. mongolicus mitogenome*, fragments transferred from chloroplasts to mitochondria.
